# The Role of Autologous Stem-Cell Transplantation in High-Risk Neuroblastoma Consolidated by anti-GD2 Immunotherapy. Results of Two Consecutive Studies

**DOI:** 10.3389/fphar.2020.575009

**Published:** 2020-10-30

**Authors:** Jaume Mora, Alicia Castañeda, Miguel A. Flores, Vicente Santa-María, Moira Garraus, Maite Gorostegui, Margarida Simao, Sara Perez-Jaume, Salvador Mañe

**Affiliations:** Pediatric Cancer Center Barcelona, Hospital Sant Joan de Déu, Barcelona, Spain

**Keywords:** neuroblastoma, autologous stem cell transplantation, overall survival, event-free survival, sargramostim, anti-GD2 antibodies

## Abstract

**Background:** Treatment of HR-NB comprise induction, consolidation with autologous stem cell transplant (ASCT) followed by anti-GD2 immunotherapy and isotretinoin. Childrens Oncology Group and SIOPEN studies used dinutuximab and cytokines to treat patients in complete remission or refractory Bone/Bone marrow (B/BM) disease after ASCT.

**Methods:** HR-NB patients referred to Hospital Sant Joan de Déu for anti-GD2 immunotherapy were eligible for two consecutive studies (dinutuximab for EudraCT 2013–004864–69 and naxitamab for 017–001829–40) and naxitamab/Sargramostim CU with or without prior ASCT. Patients enrolled in first complete remission or with primary refractory B/BM disease. We accrued a study population of two groups whose therapy, aside from ASCT, was similar. This is a retrospective analysis of their outcome calculated from study entry.

**Results:** From December 2014–2019, 67 patients were treated with dinutuximab and cytokines (n = 21) in the Hospital Sant Joan de Déu-HRNB-Ch14.18 study or with naxitamab and Sargramostim either in the Ymabs study 201 (n = 12) or CU (n = 34). 23 patients were treated with primary refractory disease in the B/BM (11 with dinutuximab and 12 with naxitamab), and 44 in first CR (10 with dinutuximab and 34 with naxitamab). Study patients included 13 (19.4%) treated following single ASCT and 54 following conventional chemotherapy. Median follow-up for all patients is 16.2 months. Two-year rates for ASCT and non-ASCT patients were, respectively, EFS 64.1% vs. 54.2% (*p* = 0.28), and OS 66.7% vs. 84.1% (*p* = 0.81). For the 44 pts in first CR, 2-years rates for ASCT and non-ASCT patients were, respectively, EFS 65.5% vs. 58.7% (*p* = 0.48), and OS 71.4% vs. 85.4% (*p* = 0.63).

**Conclusions:** In this retrospective, single center study, ASCT did not provide survival benefit when anti-GD2 immunotherapy was used after induction chemotherapy.

## Introduction

High-risk neuroblastoma is defined by metastatic disease over the age of 18 months or MYCN amplification at any age (Fletcher et al., 2018). Treatment approaches within the major international cooperative groups (GPOH, COG and SIOP) comprise a backbone of intensive induction, consolidation with high dose chemotherapy and ASCT, and isotretinoin as maintenance therapy. The most recent report from a cooperative group study with this classical approach (pre anti-GD2 immunotherapy era) is from the GPOH trial NB 2004 (Berthold et al., 2020). This study run from 2004 until 2016 and reports a 3-years EFS and OS of ≈33% and 52%, respectively.

More recently, anti-GD2 based immunotherapy was introduced in the treatment backbone and is now part of the standard of care for HR-NB. In 2010 the results of the COG ANBL0032 trial were published demonstrating that the 2-years EFS and OS of patients with HR-NB achieving major responses (after standard induction and consolidation -including ASCT-) and receiving anti-GD2 immunotherapy (dinutuximab and cytokines: GM-CSF and IL-2) in addition to isotretinoin were significantly higher (2-y EFS 66 ± 5% and OS 86 ± 4%) compared to those patients receiving isotretinoin alone (2-y EFS 46 ± 5% and OS 75 ± 5%) (Yu et al., 2010). This study is the first and only randomized trial in NB showing significant improvement in OS (Ding et al., 2018).

In 2009 the SIOPEN group designed a randomized trial to allow HR-NB patients to receive the anti-GD2 antibody dinutuximab beta with or without IL-2 with all patients receiving oral RA. The trial showed that the addition of SC IL-2 to immunotherapy with dinutuximab beta did not improve outcome, but increased toxicity (Ladenstein et al., 2018). The SIOPEN cohort analysis also showed superior EFS and OS when dinutuximab beta based immunotherapy was included compared to the same trial when RA alone was the only element of maintenance therapy. The SIOPEN study included patients receiving ASCT within 9 months from diagnosis and no progression in 109 days. Only patients without progressive disease at this time point were included in the pre-immunotherapy control population. Patients treated in the immunotherapy cohort had 2 y-EFS of 68% compared to 54% in the pre-immunotherapy cohort. Similar to the COG study, the SIOPEN trial included patients with residual disease at the site of the primary tumor or MIBG positive skeletal disease (as we defined primary refractory disease in the B/BM compartment). An improved response was found following immunotherapy in this subgroup. A 49% response rate and 40% CR rate was observed in patients treated with immunotherapy compared to 36% overall response and 32% CR rate in the control population (Ladenstein et al., 2020).

Since December 2014, HR-NB patients referred to HSJD for anti-GD2 immunotherapy were eligible whether they had received ASCT or not. The non-ASCT strategy was based upon prior experience from MSKCC showing similar outcomes for HR-NB patients managed with dose-intensive induction regimens, anti-GD2 plus GM-CSF based immunotherapy, and RT to the primary site (Kushner et al., 2016). For the last 5 years, we accrued a study population of two groups treated during the same period and whose consolidative therapy, aside from ASCT, was similar: anti-GD2 (dinutuximab or naxitamab) monoclonal antibodies + GM-CSF and local RT for all patients. In this report, we aim to assess the contribution of ASCT to outcome for patients with HR-NB. We investigate the survival of patients with the same eligibility criteria treated with ASCT or without ASCT. We analyzed this experience statistically to learn if ASCT improved prognosis.

## Materials and Methods

We report on patients treated in our center with anti-GD2 based immunotherapy from December 1st, 2014 until December 31st, 2019. Eligibility criteria included all patients with HR-NB (stage 4 at age >18 months or MYCN-amplified stage 2/3/4 at any age) to consolidate first complete remission (n = 44) or primary refractory B/BM disease (n = 23) documented following ASCT or induction regimens including chemotherapy and surgery. We define primary refractory disease as incomplete response (persistent detectable disease) in the bone and/or BM to chemotherapy induction regimens that included at least five cycles of chemotherapeutic agents including alkylators and platinum-containing compounds. Sequentially, patients were enrolled on protocol HSJD HR-NB-ch14.18 (supported by United Therapeutics, run from December 1st, 2014 until May 2017) with dinutuximab (EudraCT 2013–004864–69; n = 21), and the successor protocol Ymabs 201 (Sponsored by Ymabs Therapeutics, run from June 2017 until December 2019) with naxitamab (EudraCT 017–001829–40; n = 12) for primary refractory patients in the B/BM compartment, or CU of naxitamab and GM-CSF for patients in first complete remission (n = 34).

Patients were eligible for immunotherapy treatment if major organ toxicity was grade <2 by Common Terminology Criteria for Adverse Events Version 4.0. Informed written consents for treatments and tests were obtained according to HSJD institutional review board rules.

### Immunotherapy Treatment

The dinutuximab-based treatment followed the same guidelines as the COG ANBL0032 protocol (Yu et al., 2010). Patients received five courses of dinutuximab + GM-CSF + IL-2 at intervals of 28 days for all courses. Dinutuximab treatment was inpatient administered at 17.5 mg/m^2^/day for 4 days. Each dose of dinutuximab was infused IV over 10–20 h, starting at 0.875 mg/m^2^/h × 0.5 h then increased to 1.75 mg/m^2^/h for the remainder of the dose if tolerated. GM-CSF was administered at 250 μg/m^2^/day SC daily from day 0–13 (daily with the infusion of dinutuximab and for 3 days before and 7 days after) for cycles 1, 3, and 5. IL-2 at 3 MIU/m^2^/day was given by continuous infusion for 4 days during the first week of each course two and four on days 0–3, and at 4.5 MIU/m^2^/day on days 7–10 with the infusion of dinutuximab, cycles 2 and 4.

Naxitamab-based immunotherapy cycles comprised priming doses of SC GM-CSF for 5 days at 250 μg/m2/day (days -4 to 0), followed by naxitamab + SC GM-CSF for 5 days at 500 μg/m2/day (days 1–5). Naxitamab was infused IV over 30 min, at 3 mg/kg/day on days 1, 3, and 5 for a total dose of 9 mg/kg per cycle. GM-CSF was not given if the ANC was >20,000/μL. Treatment cycles were repeated every 4 weeks (±1 week) for a total of five cycles or until CR followed by five additional cycles every 4 weeks (±1 week). Naxitamab treatment was outpatient in all cases.

All patients who completed ≥2 courses of therapy were evaluable for inclusion in the response analysis.

### Patient Evaluations

Disease status was assessed at study entry by histology of BM aspirates obtained from bilateral posterior and bilateral anterior iliac crests, MIBG scan, and whole body MRI. PET-FDG was used for MIBG non-avid cases at diagnosis. Disease response was defined according to the revised INRC (Park et al., 2017). Four BM aspirates and ^123^I-MIBG/PET-FDG scans were performed every two to three cycles in all patients to assess response. Treatment could be continued for a response of SD or better for up to four courses. After four courses of treatment, patients with at least PR could continue treatment for up to a total of 10 cycles, provided that they did not have disease progression or drug intolerance.

Quantitative reverse transcription-polymerase chain reaction was used to assess MRD, as described (Mora et al., 2015), in BM before treatment and after every two cycles of immunotherapy. During follow-up, disease status was assessed every 3 months for 2 years by histology of BM aspirates (x4) and MRD plus ^123^I-MIBG/PET-FDG scans, and once a year craniospinal MRI.

### Statistical Analysis

Continuous variables were described using the median, minimum, and maximum. Categorical variables were described using absolute frequencies and percentages. EFS was defined as time from start of immunotherapy to PD, relapse, secondary malignancy, or toxic death, and was censored at last follow-up in the absence of these events. OS was defined as time from start of immunotherapy to death and was censored at last follow-up if death did not occur. The Kaplan-Meier method (Kaplan and Meier, 1958) was used to estimate the probability of EFS and OS. Prognostic impact of clinical and biological features on EFS and OS was tested by the log-rank test (Kalbfleisch and Prentice, 1980). Optimal cut-offs for age were estimated using the Contal-O’Quigley method (Contal and O’Quigley, 1990) with OS as outcome.

## Results

### Patient Characteristics

Sixty-seven patients met the inclusion criteria and were consecutively enrolled (Dec/2014 – Dec/2019), 21 in the dinutuximab study (United Therapeutics sponsored EudraCT 2013–004864–69) and 12 in the naxitamab study (Ymabs Therapeutics EudraCT 017–001829–40) or naxitamab CU program (n = 34). Forty-four patients (65.7%) were in first complete remission at the time of enrollment and 23 (34.3%) had primary refractory disease in the B/BM compartment by MIBG and/or conventional cytomorphology examination of the BM. All but one patient had stage 4 disease and median age at study entry is 3.6 years. The only stage 3 case was MYCN non-amplified but c-Myc overexpressed and with biological profile of high-risk (Garcia et al., 2012). Twelve (18.2%) patients had MYCN amplified neuroblastoma. Median follow-up from study entry is 16.2 months. Table 1 summarizes the clinical characteristics of the study patients and Supplementary Table S1 provides the details for each patient of all prior treatments received before immunotherapy.

**TABLE 1:** Summary descriptives for all patients. Qualitative variables are shown in absolute frequencies (percentages); quantitative variables shown as median [minimum; maximum].

	*n*
Type of patient: 67
1st CR	44 (65.7%)
Primary refractory	23 (34.3%)
Gender: 67
Female	29 (43.3%)
Male	38 (56.7%)
Age at diagnosis (years): 67	3.6 [1.2;13.5]
Stage: 67
3	1 (1.5%)
4	66 (98.5%)
MYCN: 66
Amplified	12 (18.2%)
Not amplified	54 (81.8%)
Chemo cycles: 67
5 or less	14 (20.9%)
More than 5	53 (79.1%)
Previous ASCT: 67
No	54 (80.6%)
Yes	13 (19.4%)
Previous RT: 67
No	27 (40.3%)
Yes	40 (59.7%)
MRD: 67
No	51 (76.1%)
Yes	16 (23.9%)
Dinutuximab: 67
No	46 (68.7%)
Yes	21 (31.3%)

Fifty-four patients (80.6%) were referred for anti-GD2 immunotherapy following induction chemotherapy and surgery and 13 (19.4%) after one round of high-dose chemotherapy and ASCT. Eleven of the 13 patients treated after ASCT were in first CR and two had primary refractory B/BM disease. Supplementary Table S2 provides the comparison of the clinical characteristics between the ASCT vs non- ASCT groups showing no statistical differences.

### Survival/Outcome

**FIGURE 1:**
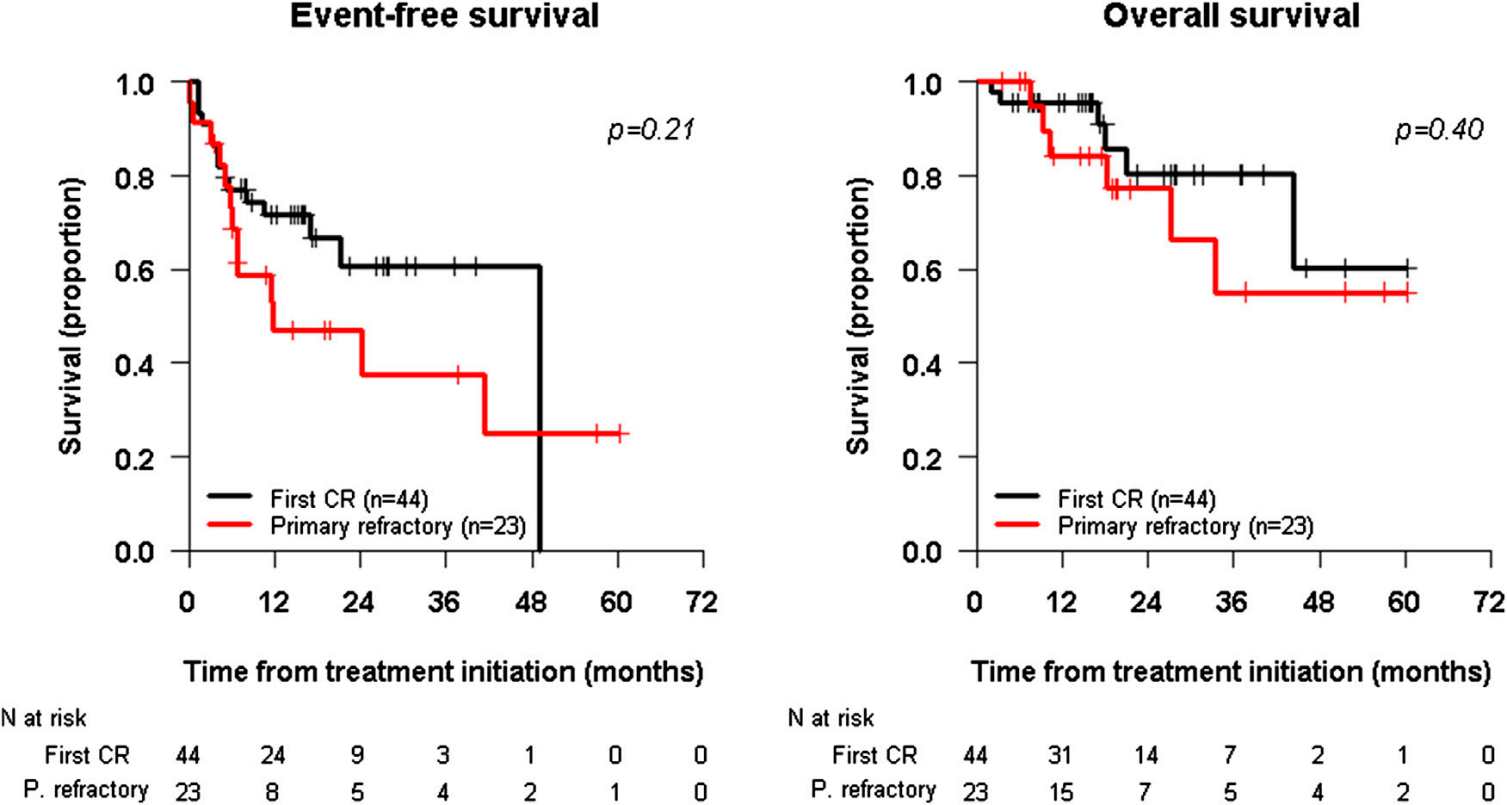
Kaplan-Meier survival curves. EFS in the Left panel and OS in the Right panel. Survival curve for patients treated with anti-GD2 immunotherapy in first complete remission (CR) is depicted in Black Line and for patients treated with primary refractory disease to the osteomedullary (Bone/Bone Marrow) compartment is shown in Red Line. Survival curves are defined from the time of immunotherapy initiation.

The 2-years EFS and OS for the whole population (n = 67) of the study was 56.6% [95% CI 44.2–72.5%] and 79.2% [95% CI 67.4–93.2%], respectively. The 2-years EFS was 47.1% [95% CI 29.4–75.3%] for primary refractory patients (n = 23) compared to 60.7% [95% CI 44.95–82.0%] for first complete remission patients (n = 44) (*p* = 0.21). The 2-years OS for the primary refractory cohort was 77.2% [95% CI 59.6–100.0%] compared to 80.2% [95% CI 65.1–98.89%] for first complete remission patients (Figure 1).

**FIGURE 2:**
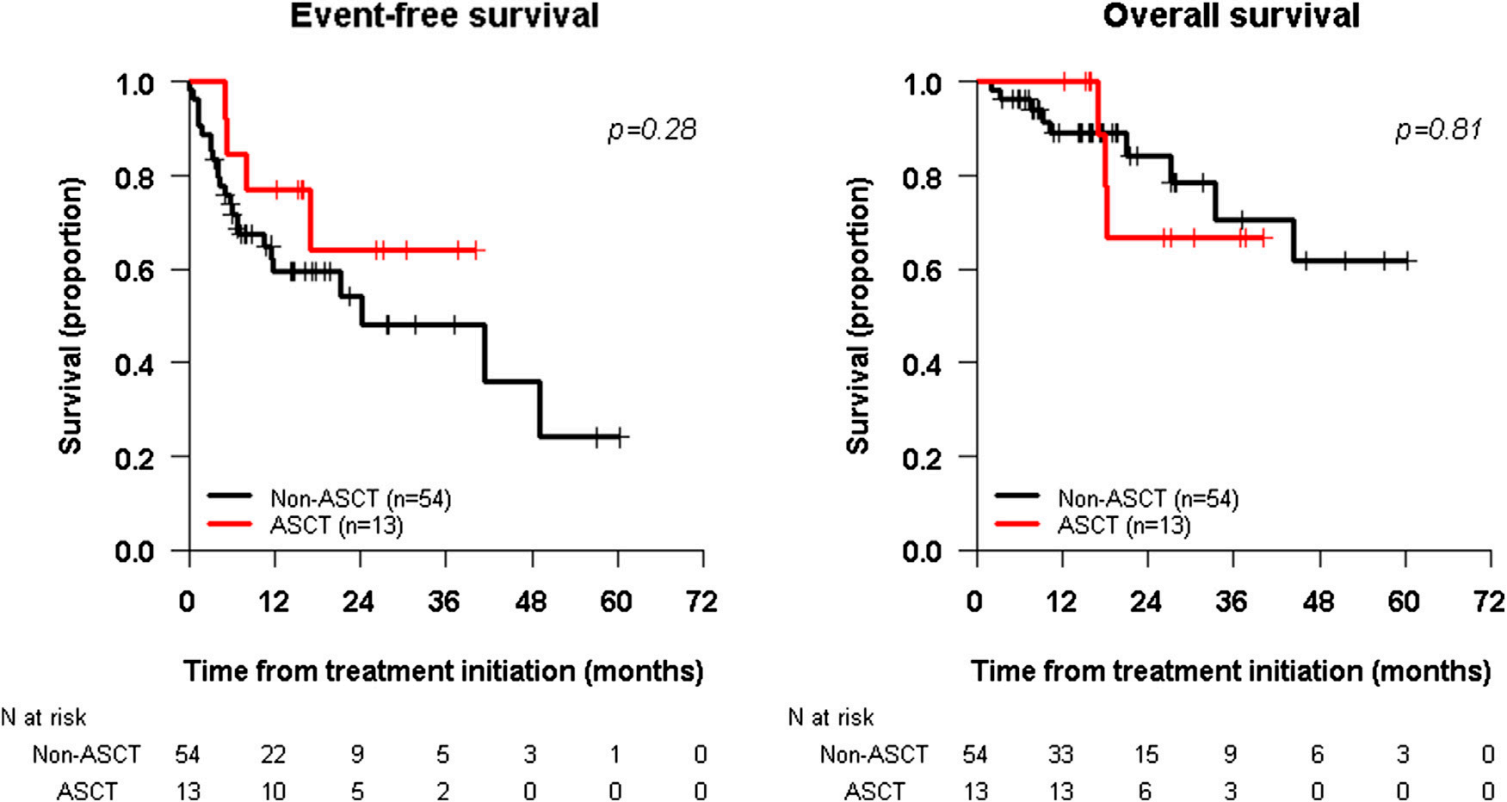
Kaplan-Meier survival curves, EFS in the left panel and OS in the Right panel, for two subgroup of patients: those who received ASCT in their consolidation regimen **(Red Line)** and those who did not receive ASCT prior to immunotherapy **(Black Line)**. Survival curves are defined from the time of immunotherapy initiation.

Two-year rates for the ASCT cohort (n = 13) and the non-ASCT cohort (n = 54) were, respectively: EFS 64.1% (95% CI: 40.2%–100%) vs. 54.2% (95% CI: 40.1%–73.2%) (log-rank *p* = 0.28), and OS 66.7% (95% CI: 42.0%–100%) vs. 84.1% (95% CI: 72.2%–97.9%) (log-rank *p* = 0.81) (Figure 2) with median follow-up for ASCT patients of 26.7 and 16.0 months for the non-ASCT cohort. Excluding the 23 primary refractory patients, two-year survival rates for the ASCT cohort (n = 11) vs the non-ASCT cohort (n = 33) were, respectively: EFS 65.5% (95% CI: 38.9%–100%) vs. 59.7% (95% CI: 40.9%–86.3%) (*p* = 0.48), and OS 71.4% (95% CI: 44.7%–100%) vs. 85.4% (95% CI: 69.5%–100%) (*p* = 0.63).

## Influence of Risk Factors

Disease status and treatment prior to anti-GD2 immunotherapy was analyzed. Variables like gender, age at diagnosis, complete remission status, MYCN, number of induction chemotherapy cycles, ASCT, MRD status and RT were included (Table 2). Older age (>4 years) and female gender were associated with lower EFS in the analysis population, but not OS. Table 3 summarizes the 2-years survival and log-rank tests for first complete remission patients only.

**TABLE 2:** 2-years survival, 95% confidence intervals and log-rank test for EFS and OS for all variables analyzed, for the whole cohort of patients.

		*n*	EFS				OS			
			2-year survival	95% CI		*p*-value	2-year survival	95% CI		*p*-value
	All patients	67	56.6	44.2	72.5	—	79.2	67.4	93.2	—
Type	1st CR	44	60.7	44.9	82.0	0.21	80.2	65.1	98.8	0.40
	Primary refractory	23	47.1	29.4	75.3		77.2	59.6	100.0	
Gender	Female	29	44.0	27.0	71.4	0.023	76.2	59.1	98.3	0.50
	Male	38	66.6	51.4	86.3		82.2	67.0	100.0	
Age at diagnosis	<4 years	42	73.3	60.9	88.2	0.013	78.3	63.6	96.4	0.26
	≥4 years	25	20.6	6.7	63.9		81.0	62.8	100.0	
MYCN	Amplified	12	50.0	28.4	88.0	0.35	50.9	24.6	100.0	0.088
	Not amplified	54	58.5	44.8	76.5		85.7	74.1	99.0	
Cycles	5 or less	14	40.0	20.2	79.2	0.12	92.9	80.3	100.0	0.55
	More than 5	53	63.1	49.9	79.7		72.1	56.2	92.4	
Previous ASCT	No	54	54.2	40.1	73.2	0.28	84.1	72.2	97.9	0.81
	Yes	13	64.1	40.2	100.0		66.7	42.0	100.0	
Previous RDT	No	27	58.6	37.3	91.9	0.55	91.2	80.2	100.0	0.65
	Yes	40	54.8	40.5	74.2		75.6	60.9	93.9	
MRD	No	51	53.9	39.6	73.3	0.58	73.3	58.9	91.1	0.21
	Yes	16	64.6	43.6	95.8		100.0	100.0	100.0	

**TABLE 3:** 2-years survival, 95% confidence intervals and log-rank test for EFS and OS for all variables analyzed. First CR patients only.

		*n*	EFS				OS			
			2-year survival	95% CI		*p*-value	2-year survival	95% CI		*p*-value
	All patients	44	60.7	44.9	82.0	—	80.2	65.1	98.8	—
Gender	Female	19	44.9	24.6	82.1	0.056	79.5	60.2	100.0	0.60
	Male	25	74.0	55.1	99.2		79.5	57.7	100.0	
Age at diagnosis	< 1.6 years	5	80.0	51.6	100.0	0.48	100.0	100.0	100.0	0.26
	≥ 1.6 years	39	57.2	40.0	81.8		76.5	59.3	98.8	
MYCN	Amplified	10	60.0	36.2	99.5	0.58	56.3	27.9	100.0	0.12
	Not Amplified	34	61.6	43.5	87.2		89.6	75.8	100.0	
Cycles	5 or less	10	45.0	21.1	96.1	0.36	90.0	73.2	100.0	0.73
	More than 5	34	68.6	52.2	90.0		72.1	50.9	100.0	
Previous ASCT	No	33	58.7	39.9	86.3	0.48	85.4	69.5	100.0	0.63
	Yes	11	65.5	38.9	100.0		71.4	44.7	100.0	
Previous RT	No	23	63.1	40.7	97.8	0.58	95.7	87.7	100.0	0.37
	Yes	21	58.3	39.1	87.0		69.8	48.4	100.0	
MRD	No	38	58.9	41.4	83.8	0.87	77.5	61.0	98.6	0.38
	Yes	6	66.7	37.9	100.0		100.0	100.0	100.0	

## Toxicity

Three (14.3%) out of the 21 patients treated in the dinutuximab protocol did not complete planned therapy because of ≥ grade 4 toxicity. Two were previously reported with transverse myelitis (Ding et al., 2018) and one developed severe macrophage activated syndrome for which the patient eventually died. Four (8.7%) of the 46 patients treated with naxitamab and GM-CSF did not complete planned cycles because of severe toxicity. Three because of grade 4 apnea at the initiation of antibody infusion that was not manageable with advanced supportive care. One patient had a stroke between antibody infusions and although was deemed not related to antibody, was taken off the study.

## Discussion

Recently, clinical trials of GD2-targeting monoclonal antibody therapy have demonstrated significantly prolonged EFS and, more importantly, OS when used after first complete or very good partial response to standard therapy, leading to regulatory approval in the United States and Europe (Yu et al., 2010; Ladenstein et al., 2018). The only randomized controlled trial included 226 participants with HR-NB who were randomized to receive either dinutuximab-containing immunotherapy or isotretinoin after pre-treatment with high-dose chemotherapy followed by ASCT (ANBL0032). The primary objective of ANBL0032 was an intention-to-treat comparison of EFS in the two treatment groups. Authors provided statistical testing at 2 years and estimated an OS of 86% in the dinutuximab-containing immunotherapy group and 75% in the isotretinoin group, resulting in a *p* value of 0.02. At 2 years they estimated an EFS of 66% in the dinutuximab-containing immunotherapy arm and an EFS of 46% in the standard therapy group, resulting in a *p* value of 0.01. In 2014 associated with the ANBL0032 study, an abstract provided updated data on survival, and as opposed to the original publication, the difference between the treatment groups was no longer statistically significant for EFS. For the immunotherapy cohort EFS was 67 ± 4% at 2-years and 59 ± 5% at 4-years vs. 51 ± 5% (2-years) and 48 ± 5% (4-years) for the isotretinoin cohort (*p* = 0.11). Importantly, the 4-years OS rate remained significantly improved (*p* = 0.02) with immunotherapy (Yu et al., 2014). Meantime, in Europe the HR-NBL1/SIOPEN trial randomized patients to receive either dinutuximab-beta and SC IL-2 with isotretinoin or dinutuximab-beta with isotretinoin, to investigate the role of IL-2 (Ladenstein et al., 2018). The three-year EFS was 56% with dinutuximab-beta and 60% with dinutuximab-beta and SC IL-2 (*p* = 0.76). Furthermore, the SIOPEN study authors showed that the 2-years EFS with dinutuximab-beta was comparable with the 2-years EFS previously reported by the COG with dinutuximab. In this study, we report a similar range of 2-years EFS of 54.2% and OS of 84.1% in a cohort of HR-NB patients having achieved first complete remission or very good partial response and consolidated without prior ASCT.

Therapy in all major cooperative studies includes ASCT, since approval of anti-GD2 antibodies was subjected to what was considered “standard” at the time. Among the components of “standard therapy” for HR-NB, ASCT has been repeatedly questioned since prior experiences showed an uncertain benefit of ASCT (Ladenstein et al., 2008; Yanik et al., 2013). The landmark CCG study 3891 primary question was whether ABMT improved EFS at three years from diagnosis for children with HR-NB. The CCG study randomized patients to receive a conditioning regimen of carboplatin, etoposide, melphalan and total-body irradiation followed by an infusion of purged BM vs continuation-chemotherapy of three cycles of cisplatin, etoposide and doxorubicin with ifosfamide (Matthay et al., 1999). The study results were corrected several years later by the COG showing no OS advantage with ABMT. The corrected *p*-value for OS at 5-years for ABMT vs. continuing chemotherapy was *p* = 0.08 and not *p* < 0.0001 (Matthay et al., 2009). In 2013, a Cochrane meta-analysis clearly showed that ABMT/ASCT did not improve OS for HR-NB (Yalcin et al., 2013). More recently, a detailed analysis by the SIOPEN group of risk factors and the relevance for each of the sequential treatment interventions (chemotherapy, ASCT, immunotherapy) that composes HR-NB multimodal treatment, has revealed that the impact of immunotherapy on EFS is significantly influenced by variables like stage, pattern of metastases, but is borderline related (suggesting interaction) to ASCT (Ladenstein et al., 2020). This interaction generates an interesting biology question for future studies.

Prior experience suggested tandem transplant is not helpful for HR-NB. In the sole reported randomized trial involving tandem transplant (Park et al., 2019), comparison was with single transplant (not with no transplant): tandem showed better EFS but OS was again not significantly different (Park et al., 2019). The fundamental question as to whether transplant - single or double–is warranted for HR-NB remains unanswered.

EFS is a surrogate endpoint for early assessment of efficacy, but its validity requires confirmation, either through association with OS or by meta-analysis (Ellis et al., 2014). In the corrected randomized CCG study (Matthay et al., 2009) there was no OS advantage with ABMT for HR-NB thus improvement of EFS did not associate with better survival, the most relevant outcome measure for patients (Ellis et al., 2014). On the other hand, the impact of anti-GD2 immunotherapy on OS is significant and recurrent in all reported studies (Yu et al., 2010; Ladenstein et al., 2018; Ladenstein et al., 2020; Kushner et al., 2016). This is especially relevant for patients and families. Even in our limited cohort of ASCT patients, better EFS (64%) was shown for the ASCT cohort compared to the non-ASCT (54%), although not statistically significant. However, for OS the ASCT group showed an inferior (66%) rate compared to the non-ASCT (84%) group. Of note is that OS takes into account safety (toxic complications) which is a major concern with transplant procedures (Martin et al., 2014). Even more importantly, improvement in OS also reflects a novel paradigm in the anti-GD2 immunotherapy era: relapse is no longer an invariably lethal event. In the current era of anti-GD2 immunotherapy relapses occur more limited, isolated, less aggressive, and more amenable to control (Kushner et al., 2015; Mody et al., 2017; Mody, 2020). Further evidence of the overall survival benefit resulting from anti-GD2 immunotherapy is the outcome of the refractory cohort in this study and others (Kushner et al., 2015; Mody et al., 2017; Mody, 2020).

In our experience, HR-NB patients whose therapy of first complete remission or primary refractory B/BM disease included anti-GD2 antibodies and GM-CSF had similar survival outcomes whether these were administered following ASCT or conventional induction treatment. Since most HR-NB patients are treated with protocols that include ASCT, accruing a significant number of cases managed without high-dose chemotherapy and ASCT is currently uncommon. Our set of patients adds to a larger and independent cohort from MSKCC (Kushner et al., 2016), increasing the population of HR-NB patients in first complete remission or with primary refractory B/BM disease managed in the current era of anti-GD2 immunotherapy without ASCT and showing statistically no different survival rates as those who have received ASCT. Advances in anti-GD2 immunotherapy like the use of more potent and/or higher doses of anti-GD2 antibodies, the lesser use of IL-2 that proved no beneficial (Ladenstein et al., 2018), and the systematic use of GM-CSF may account for the lack of survival advantage of ASCT in this current era.

The limitations of this study include the single institution experience, the retrospective review of the data including potential confounding variables like different induction regimens and myeloablative therapies. Taking into account all the previous limitations, however, our results suggest that ASCT may not improve outcome when current regimens including anti-GD2 antibodies in combination with GM-CSF are used for treating HR-NB patients in first complete remission or primary refractory B/BM disease.

## Data Availability Statement

The raw data supporting the conclusions of this article will be made available by the authors, without undue reservation.

## Ethics Statement

The studies involving human participants were reviewed and approved by Hospital Sant Joan de Deu. Written informed consent to participate in this study was provided by the participants' legal guardian/next of kin.

## Author Contributions

AC, MF, VS-M, MG, MG, MS, and SM conducted the clinical trials and secured patient data management. SP-J conducted the biostatistical analysis. JM supervised the overall study and wrote the manuscript. All authors revised the final manuscript and approved its final format.

## Conflict of Interest

JM declare consulting fees from United Therapeutics and Ymabs Therapeutics.

The remaining authors declare that the research was conducted in the absence of any commercial or financial relationships that could be construed as a potential conflict of interest

## Glossary

ABMT: Autologous bone Marrow transplant
ANC: Absolute Neutrophil Count
ASCT: Autologous stem Cell transplant
B/BM: Bone/Bone marrow
CCG: Childrens Cancer group
COG: Childrens Oncology Group
CR: Complete remission
CU: Compassionate use
EFS: Event-Free survival
GD2: Disialoganglioside
GM-CSF: Sargramostim
GPOH: German Pediatric Oncology and Hematology
HSJD: Hospital Sant Joan de Déu
HR-NB: High-risk neuroblastoma
IL-2: Interleukin-2
INRC: International neuroblastoma response Criteria
IV: Intravenous
MIBG: ^123I^-Metaiodobenzylguanidine
MRD: Minimal residual disease
MRI: Magnetic Resonance Imaging
OS: Overall survival
PET-FDG: Fluorodeoxyglucose (FDG)-positron emission tomography (PET)
PR: Partial response
RA: Retinoic Acid
RT: Radiation therapy
SC: Subcutaneous
SD: Stable disease
SIOP: Societe Internationale d'Oncologie Pediatrique
