# Supplementary material for: The Role of Autologous Stem-Cell Transplantation in High-Risk Neuroblastoma Consolidated by anti-GD2 Immunotherapy. Results of Two Consecutive Studies
Source: Front Pharmacol. 2020 Oct 30;11:575009. doi: 10.3389/fphar.2020.575009 (PMC7723438; doi:10.3389/fphar.2020.575009)
Supplement: Supplementary file 1 [file Table1_v1.docx]

Supplementary Table 1:

| **CR pts** | **Ab** | **Stage** | **MYCN** | **Induction Protocol** | **ASCT** | **Relapse** | **Status** |
| --- | --- | --- | --- | --- | --- | --- | --- |
| 1 | DINU | 4 | A | GPOH | Bu/Mel | Y | DOD |
| 2 | DINU | 4 | NA | COJEC | N | Y | NED |
| 3 | DINU | 4 | NA | mN7 | N | N | DOD |
| 4 | DINU | 4 | NA | mN7 | N | Y | DOD |
| 5 | DINU | 4 | NA | mN7 | N | Y | NED |
| 6 | DINU | 4 | A | mN7 | N | Y | AWD |
| 7 | DINU | 4 | NA | mN7 | N | N | NED |
| 8 | DINU | 4N | A | COJEC | Bu/Mel | N | NED |
| 9 | DINU | 4 | A | COJEC | Bu/Mel | Y | DOD |
| 10 | DINU | 4 | NA | GPOH | CEM | Y | NED |
| 1 | NAXI | 4 | A | mN7 | N | N | NED |
| 2 | NAXI | 4 | NA | mN7 | N | Y | DOD |
| 3 | NAXI | 4 | NA | GPOH | CEM | N | NED |
| 4 | NAXI | 4 | NA | mN7 | N | N | NED |
| 5 | NAXI | 4 | NA | COJEC | N | N | NED |
| 6 | NAXI | 4 | NA | GPOH | CEM | N | NED |
| 7 | NAXI | 4 | NA | mN7 | N | Y | NED |
| 8 | NAXI | 4 | NA | COG | CEM | N | NED |
| 9 | NAXI | 4 | NA | CCCG NB2014 | N | N | NED |
| 10 | NAXI | 4 | A | COG | N | N | NED |
| 11 | NAXI | 4 | NA | CCCG NB2014 | N | N | NED |
| 12 | NAXI | 4 | NA | mN7 | N | N | NED |
| 13 | NAXI | 4 | NA | CCCG NB2014 | N | N | NED |
| 14 | NAXI | 4 | NA | CCCG NB2014 | N | Y | NED |
| 15 | NAXI | 4 | NA | CCCG NB2014 | Bu/Mel | N | NED |
| 16 | NAXI | 4 | NA | CCCG NB2014 | Bu/Mel | N | NED |
| 17 | NAXI | 4 | NA | COJEC | Bu/Mel | N | NED |
| 18 | NAXI | 4 | NA | N7 | N | N | NED |
| 19 | NAXI | 4 | NA | mN7 | N | N | NED |
| 20 | NAXI | 4 | NA | mN7 | N | N | NED |
| 21 | NAXI | 4 | NA | CCCG NB2014 | N | N | NED |
| 22 | NAXI | 4 | A | CCCG NB2014 | N | Y | DOD |
| 23 | NAXI | 4 | A | COJEC | Bu/Mel | N | NED |
| 24 | NAXI | 4 | A | CCCG NB2014 | N | N | NED |
| 25 | NAXI | 4 | A | CCCG NB2014 | N | N | NED |
| 26 | NAXI | 4 | NA | CCCG NB2014 | N | Y | NED |
| 27 | NAXI | 4 | NA | CCCG NB2014 | N | Y | NED |
| 28 | NAXI | 4 | NA | CCCG NB2014 | N | N | NED |
| 29 | NAXI | 4 | NA | COG | N | Y | NED |
| 30 | NAXI | 4 | NA | CCCG NB2014 | N | N | NED |
| 31 | NAXI | 4 | NA | CCCG NB2014 | N | N | NED |
| 32 | NAXI | 4 | NA | CCCG NB2014 | N | N | NED |
| 33 | NAXI | 4 | NA | CCCG NB2014 | N | N | NED |
| 34 | NAXI | 4 | NA | CCCG NB2014 | N | N | NED |
| **Refractory** | **Ab** | **Stage** | **MYCN** | **Induction Protocol** | **ABMT** | **Relapse** | **Status** |
| 1 | DINU | 4 | NA | mN7 | Bu/Mel | Y | DOD |
| 2 | DINU | 3 | NA | mN7 | N | Y | DOD |
| 3 | DINU | 4 | NA | COJEC | N | Y | DOD |
| 4 | DINU | 4 | NA | mN7 | N | N | NED |
| 5 | DINU | 4 | NA | mN7 | N | Y | NED |
| 6 | DINU | 4 | NA | COJEC | Bu/Mel | N | NED |
| 7 | DINU | 4 | A | mN7 + CTV | N | Y | DOD |
| 8 | DINU | 4 | NA | mN7 + CTV | N | N | NED |
| 9 | DINU | 4 | NA | COJEC | N | Y | AWD |
| 10 | DINU | 4 | NA | COJEC | N | Y | DOD |
| 11 | DINU | 4 | NA | GPOH | N | Y | DOD |
| 1 | NAXI | 4 | NA | mN7 | N | Y | NED |
| 2 | NAXI | 4 | NA | CCCG NB2014 | N | N | NED |
| 3 | NAXI | 4 | NA | COJEC | N | Y | NED |
| 4 | NAXI | 4 | NA | CCCG NB2014 | N | N | NED |
| 5 | NAXI | 4 | NA | mN7 | N | Y | NED |
| 6 | NAXI | 4 | NA | CCCG NB2014 | N | Y | AWD |
| 7 | NAXI | 4 | NA | CCCG NB2014 | N | N | NED |
| 8 | NAXI | 4 | NA | CCCG NB2014 | N | N | AWD |
| 9 | NAXI | 4 | NA | CCCG NB2014 | N | N | NED |
| 10 | NAXI | 4 | A | N7 | N | Y | AWD |
| 11 | NAXI | 4 | NA | CCCG NB2014 | N | N | NED |
| 12 | NAXI | 4 | NA | CCCG NB2014 | N | N | AWD |
