# Supplementary material for: The Role of Autologous Stem-Cell Transplantation in High-Risk Neuroblastoma Consolidated by anti-GD2 Immunotherapy. Results of Two Consecutive Studies
Source: Front Pharmacol. 2020 Oct 30;11:575009. doi: 10.3389/fphar.2020.575009 (PMC7723438; doi:10.3389/fphar.2020.575009)
Supplement: Supplementary file 2 [file Table2_v1.docx]

**Supplementary Table** 2

|  | **Previous ASCT** | |  |
| --- | --- | --- | --- |
|  | **No** | **Yes** | **p-value** |
|  | *n=54* | *n=13* |  |
| Type of patient: |  |  | 0.19 |
| 1^st^ CR | 33 (61.1%) | 11 (84.6%) |  |
| Primary refractory | 21 (38.9%) | 2 (15.4%) |  |
| Gender: |  |  | 0.18 |
| Female | 26 (48.1%) | 3 (23.1%) |  |
| Male | 28 (51.9%) | 10 (76.9%) |  |
| Age at diagnosis (years) | 3.7 [1.2;13.5] | 2.7 [1.3;5.5] | 0.081 |
| Stage: |  |  | 1.00 |
| 3 | 1 (1.9%) | 0 (0.0%) |  |
| 4 | 53 (98.1%) | 13 (100.0%) |  |
| MYCN: |  |  | 0.23 |
| Amplified | 8 (15.1%) | 4 (30.8%) |  |
| Not Amplified | 45 (84.9%) | 9 (69.2%) |  |
| Cycles: |  |  | 0.055 |
| 5 or less | 14 (25.9%) | 0 (0.0%) |  |
| More than 5 | 40 (74.1%) | 13 (100.0%) |  |
| Previous RT: |  |  | 0.085 |
| No | 25 (46.3%) | 2 (15.4%) |  |
| Yes | 29 (53.7%) | 11 (84.6%) |  |
| MRD: |  |  | 0.16 |
| No | 39 (72.2%) | 12 (92.3%) |  |
| Yes | 15 (27.8%) | 1 (7.69%) |  |
